# Hydrodynamic Shear Effects on Grafted and Non-Grafted Collapsed Polymers

**DOI:** 10.3390/polym10080926

**Published:** 2018-08-18

**Authors:** Richard Schwarzl, Roland R. Netz

**Affiliations:** Department of Physics, Freie Universität Berlin, 14195 Berlin, Germany; rschwarz@physik.fu-berlin.de

**Keywords:** shear flow, Brownian dynamics simulation, hydrodynamic interactions, von Willebrand factor

## Abstract

We study collapsed homo-polymeric molecules under linear shear flow conditions using hydrodynamic Brownian dynamics simulations. Tensile force profiles and the shear-rate-dependent globular-coil transition for grafted and non-grafted chains are investigated to shine light on the different unfolding mechanisms. The scaling of the critical shear rate, at which the globular-coil transition takes place, with the monomer number is inverse for the grafted and non-grafted scenarios. This implicates that for the grafted scenario, larger chains have a decreased critical shear rate, while for the non-grafted scenario higher shear rates are needed in order to unfold larger chains. Protrusions govern the unfolding transition of non-grafted polymers, while for grafted polymers, the maximal tension appears at the grafted end.

## 1. Introduction

The von Willebrand factor (VWF) is a large glycoprotein that is crucially involved in primary hemostasis [1]. The ability to mediate platelet adhesion at sites of vascular injury depends on the activation stage of VWF [2,3,4] and its insufficiency has been linked to bleeding disorders [5]. The proposed mechanism for the activation of the VWF is the elongation and partial unfolding by elevated shear flow conditions due to vasoconstriction [3,6,7,8]. This implicates a collapsed state of VWF present under normal shear flow conditions and a shear-flow induced transition into a partly unfolded state [7]. The behavior of collapsed as well as coiled polymers in shear flow has been at the focus of theoretical and experimental research [6,7,9,10,11,12,13]. Previous studies have used simulations and theoretical considerations to explain the globular-coil transition due to linear shear flow for collapsed polymers in the vicinity of the vessel wall [6,14,15]. Despite the fact that the grafted scenario might be even more relevant, the non-grafted scenario has been studied intensively in simulations so far. The relevance of the grafted scenario arises from the fact that upon activation, VWF binds to sub-endothelial collagen [4,16,17,18,19,20].

In the present study, we focus on the grafted scenario, which frequently appears in atomic force microscopy (AFM) measurements [4,19,21], and compare it to the non-grafted scenario. We conduct Brownian dynamics simulations including long range hydrodynamic interactions (HI) on the Rotne-Prager level [22,23] under linear shear flow conditions. Although some stationary dynamic chain properties are independent of hydrodynamic effects [24], hydrodynamic interactions crucially influence the scaling behaviour of the critical shear rate of the globular-coil transition [6,7]. These results shed light on time scales relevant for the regulatory mechanisms linked to the domain opening of VWF in the grafted scenario. One of the additional regulatory mechanisms that has been studied in more detail, is the cleavage of VWF by means of the enzyme ADAMTS-13 [25,26]. Relating simulations and results from atomic force microscopy (AFM) via rate theories [19,27,28,29,30] has been argued to offer a promising route to investigate domain specific activation times of VWF.

In the first part of this study, scaling laws for the critical shear rate are deduced from simulation results. We find the dependence of the critical shear rate on the number of monomers to be inverted for the grafted compared to the non-grafted scenario, i.e., for the grafted scenario, an increase in polymer size reduces the critical shear rate while for the non-grafted scenario the critical shear rate is increased. In the second part, we connect the unfolding of the polymer to the tensile force profile and investigate the interplay of drag and lift force that determines the configurations of the grafted chain. In addition, we investigate the mean and the maximum of the tensile force profile for the grafted and non-grafted chain at the critical shear rate. These forces and their scaling with the number of monomers are relevant for the shear-dependent folding and unfolding times of VWF domains.

## 2. Methods

### 2.1. The Model

**Figure 1:**
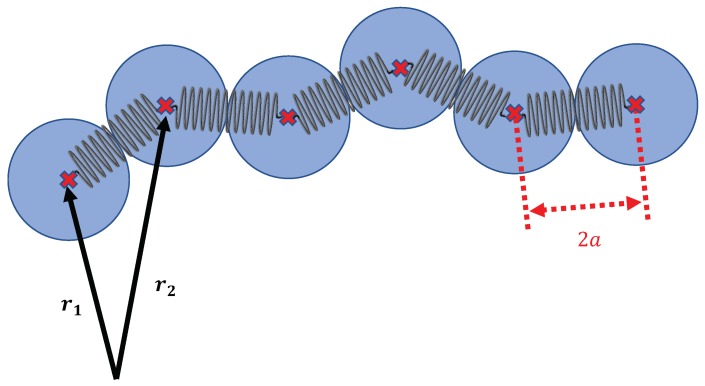
Schematic picture of the bead-spring model of the von Willebrand factor. The beads are numerated from 1 to *N* and their respective position vectors are denoted by $r_{1},\ldots ,r_{N}$. The harmonic springs connecting consecutive beads have a stiffness of $\kappa =200k_{B}{\mathrm{Ta}}^{-2}$.

We describe the VWF by a bead-spring model. The beads are numerated from 1 up to the total number of beads, *N*. Each bead has a radius of *a* which is associated with an effective radius of a VWF dimer of 73 nm [26]. A schematic description of the VWF model is given in Figure 1. The position vectors of the beads are denoted by $r_{1},\ldots ,r_{N}$. The backbone of the chain is realized by a series of springs, with a spring constant $\kappa =200k_{B}{\mathrm{Ta}}^{-2}$, that connect neighbouring beads. The potential of each spring is given as

(1) $$U_{\mathrm{SP}}\left(\right|r_{i+1}-r_{i}\left|\right)=\frac{\kappa }{2}{\left(|r_{i+1}-r_{i}|-2a\right)}^{2}.$$

All beads interact pairwise by a Lennard-Jones interaction with a cohesion strength ε, which in our study is set to $2k_{B}T$,

(2) $$U_{\mathrm{LJ}}\left(\right|r_{i}-r_{j}\left|\right)=\varepsilon \left[{\left(\frac{2a}{|r_{i}-r_{j}|}\right)}^{12}-2{\left(\frac{2a}{|r_{i}-r_{j}|}\right)}^{6}\right].$$

The value for the cohesion strength was previously obtained by fitting simulation results of the bead-spring model for the globule-to-coil transition to experimental data [7]. Note that for the relatively large VWF monomers considered here, the dispersion interaction will, for small separation, lead to a different interaction potential than the Lennard-Jones interaction given in Equation (2). We thus view Equation (2) as a model potential, in line with previous work. The total potential energy of our system follows as

(3) $$U\left(r_{1},\ldots ,r_{N}\right)=\sum_{i=1}^{N-1}\sum_{j>i}^{N}U_{\mathrm{LJ}}\left(|r_{i}-r_{j}|\right)+\sum_{i=1}^{N-1}U_{\mathrm{SP}}\left(|r_{i+1}-r_{i}|\right).$$

### 2.2. Simulation Details

We perform Brownian dynamics simulations of a single chain using a discretized version of the over-damped Langevin equation [15,22],

(4) $$\frac{r_{i}\left(t+\Delta t\right)-r_{i}\left(t\right)}{\Delta t}=\dot{\gamma }z_{i}\mu_{0}^{-1}\mu_{ii}\cdot \hat{x}-\sum_{j=1}^{N}\mu_{ij}\cdot \left[\nabla_{r_{j}\left(t\right)}U\left(r_{1},\ldots ,r_{N}\right)\right]+k_{B}T\frac{d\mu_{ii}^{zz}}{dz}{|}_{z=z_{i}}\;\hat{z}+\xi_{i}\left(t\right),$$

which is used to recursively calculate the displacement of a bead *i* in a specified time step Δt. The first term on the right-hand side of Equation (4) accounts for the linear shear flow proportional to the shear rate $\dot{\gamma }$. The direction of the flow is given by the unit vector in *x* direction, denoted as $\hat{x}$. The mobility tensor μ and random velocity $\xi_{i}$ are different for the grafted and non-grafted scenarios.

For the non-grafted situation, we use the Rotne-Prager-Yamakawa tensor to calculate the mobility of all beads. Due to the absence of a no-slip boundary in this scenario, the third term on the right side of Equation (4) vanishes. The Rotne-Prager-Yamakawa tensor is given by [31]

(5) $$\mu_{ij}=\mu^{\mathrm{RPY}}\left(r_{ij}=r_{i}-r_{j}\right)=\left\{\begin{array}{cc} \frac{1}{8\pi \eta r_{ij}}\left[\left(1+\frac{2a^{2}}{3r_{ij}^{2}}\right)1+\left(1-\frac{2a^{2}}{r_{ij}^{2}}\right){\hat{r}}_{ij}⊗{\hat{r}}_{ij}\right] & \mathrm{if}r_{ij}>2a\\ \frac{1}{6\pi \eta a}\left[\left(1-\frac{9r_{ij}}{32a}\right)1+\frac{3r_{ij}}{32a}{\hat{r}}_{ij}⊗{\hat{r}}_{ij}\right] & \mathrm{if}r_{ij}\leq 2a \end{array}\right.,$$

where $r_{ij}=\left|r_{i}-r_{j}\right|$.

For the grafted scenario which includes a no-slip boundary at z=0, we use the Rotne-Prager- Blake tensor that was previously derived [23],

(6) $$\mu_{ij}=\mu^{\mathrm{RPB}}\left(r_{i},r_{j}\right)=\mu^{\mathrm{RP}}\left(r_{i}-r_{j}\right)-\mu^{\mathrm{RP}}\left(r_{i}-{\bar{r}}_{j}\right)+\Delta \mu \left(r_{i},r_{j}\right),$$

where ${\bar{r}}_{j}={\left(x_{j},y_{j},-z_{j}\right)}^{T}$ is the mirror image position and $\mu^{\mathrm{RP}}$ is the Rotne-Prager tensor. The explicit terms of the Rotne-Prager-Blake tensor are given in the supplementary material.

The random velocity in Equation (4), $\xi_{i}$, follows from the fluctuation-dissipation theorem,

(7) $$\langle \xi_{i}\left(t\right)⊗\xi_{j}\left(t^{\prime }\right)\rangle =2k_{B}T\mu_{ij}\delta \left(t-t^{\prime }\right).$$

We use the Cholesky factorization to decompose the entire mobility matrix μ into a lower triangular matrix L and its transposed. The lower triangular matrix is then multiplied with a random Gaussian vector to obtain correlated values that obey the required variance condition given by Equation (7).

For the grafted scenario, we introduce an additional repulsive potential that prevents the beads to cross the no-slip boundary at z=0. This potential is given by [32]

(8) $$U_{R}\left(r_{i}\right)=\left\{\begin{array}{cc} \frac{2\pi k_{B}T\sigma_{R}}{a}\left[\frac{2}{5}{\left(\frac{\sigma_{R}}{z_{i}}\right)}^{10}-{\left(\frac{\sigma_{R}}{z_{i}}\right)}^{4}+\frac{3}{5}\right] & \mathrm{if}z_{i}\leq \sigma_{R}\\ 0 & \mathrm{if}z_{i}>\sigma_{R} \end{array}\right.$$

where $\sigma_{R}$ is chosen to be 1.5a. This repulsive potential makes sure that the approximation made for the parallel and perpendicular self-mobilities in the derivations of the expressions obtained by Perkins, Jones, Stimson and Jeffery remain valid (cf. von Hansen, et al., Figure 1) [23,33,34].

In simulations for the grafted scenario, the first bead is modelled as an anchor point. The position of the first bead is therefore not updated in the simulation. There is also no hydrodynamic interaction between the first bead and any other bead in the simulation. Hence the mobility matrix for this case is not 3N dimensional but rather 3(N−1) dimensional.

Simulation parameters and results are given in rescaled units. These units are the bead radius *a*, the thermal energy $k_{B}T$ and the diffusion time τ = 6πη a${}^{3}$/(*k*${}_{B}$*T*) = a${}^{2}$/($\mu_{0}$k${}_{B}$*T*). The simulation time step is consistently chosen to be Δt/τ = 5 × 10${}^{-4}$ and simulations are run for at least $2\times 10^{8}$ steps, resulting in a minimum simulation time of $10^{5}\tau$. Positions of all beads are saved at least every $10^{5}$ steps. When we calculate an observable from trajectories, we always omit the first $10^{6}$ steps for equilibration.

Throughout our simulations, we do not observe self-entanglement effects, which experimentally are known to exist for DNA [35]. Note that in similar simulations at larger cohesion strength, a non-monotonic behaviour of the chain size on shear rate has been reported and rationalized by entanglement effects [36].

## 3. Results

In our study, we systematically vary the shear rate, $\dot{\gamma }$, as well as the number of beads, *N*, of the bead-spring chain and therefore its contour length which is given as L=2(N−1)a.

### 3.1. Scaling of Critical Shear Rate

**Figure 2:**
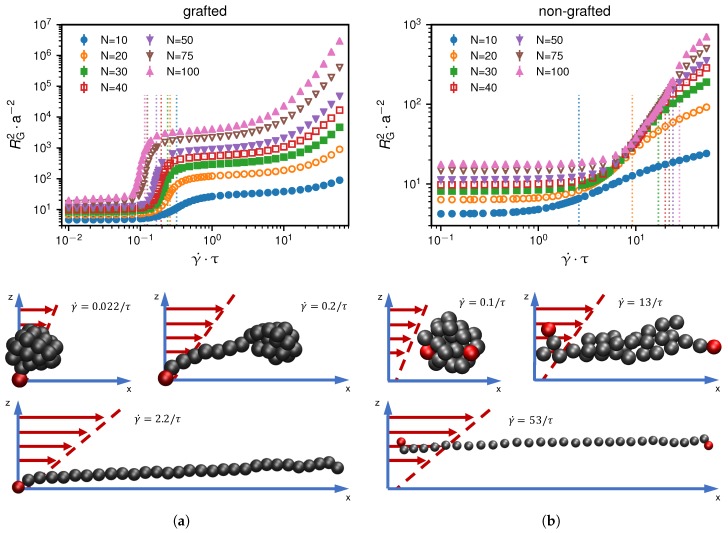
An increase of the shear rate $\dot{\gamma }$ above a certain threshold, which depends on the size of the chain, initiates a transition from a collapsed to a coiled or extended state. The vertical dashed lines relate to the steepest increase of the mean-squared radius of gyration as a function of the shear rate. We present results for different monomer numbers *N*. Subfigures (**a**,**b**) show the grafted and non-grafted cases, respectively. We also show simulation snapshots for N=30 below, at and above the critical shear rate of ${\dot{\gamma }}^{*}$ = 0.24 $\tau^{-1}$ for the grafted and ${\dot{\gamma }}^{*}$ = 13 $\tau^{-1}$ for the non-grafted scenario.

As we show in Figure 2, both the grafted as well as the non-grafted scenarios exhibit a shear-induced globule-to-coil transition. We identify this transition by calculating the mean-squared radius of gyration of the chain for different shear rates defined as

(9) $$R_{G}^{2}=\frac{1}{2N}\sum_{i=1}^{N}\sum_{j=1}^{N}{\left(r_{i}-r_{j}\right)}^{2}.$$

**Figure 3:**
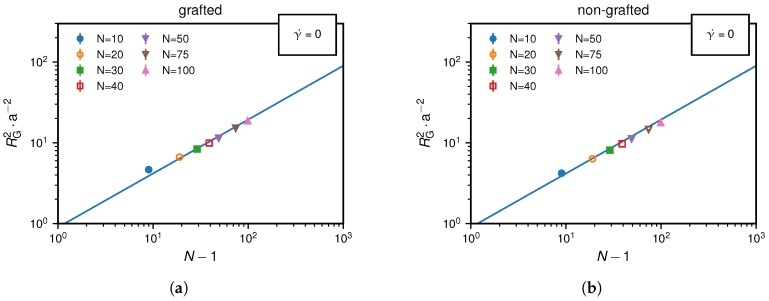
Equilibrium results for zero shear rate $\left(\dot{\gamma }=0\right)$: The mean-squared radius of gyration, $R_{G}^{2}$, as function of the monomer number, N−1, is described by the expected scaling law for collapsed polymers, $R_{G}^{2}∼{\left(N-1\right)}^{2/3}$, shown as a blue line both in (**a**) the grafted and (**b**) the non-grafted scenarios.

In Figure 2, we see a narrow shear rate range over which a significant increase of the time-averaged radius of gyration is observed. This reflects a conformational change of the bead-spring chain from a collapsed state caused by cohesion, which is verified by the scaling relation of $R_{G}^{2}∼{\left(N-1\right)}^{2/3}$ at $\dot{\gamma }=0$ shown in Figure 3, to a non-collapsed state.

Chains in shear flow show large size fluctuations [37]. To determine the transition between the collapsed and non-collapsed states, we analyse quantities that characteristically depend on the shear rate and exhibit an extremum at the transition. Previous publications used $R_{S}^{2}$, a quantity that measures the mean-squared extension of the bead-spring chain in flow-direction [14,15,26]. This quantity is defined as the maximal squared distance between any two beads after projecting their positions onto the flow-direction:

(10) $$R_{S}^{2}\left(t\right)=\max_{i,j\in \left\{1,\ldots ,N\right\}}\left[{\left(r_{ij}\cdot \hat{x}\right)}^{2}\right].$$

**Figure 4:**
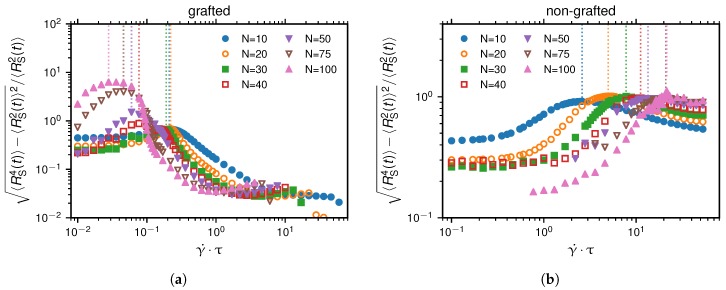
The maximum of the normalized standard deviation of the mean-squared elongation of the chain in flow direction, $\sqrt{\langle R_{S}^{4}\left(t\right)\rangle -{\langle R_{S}^{2}\left(t\right)\rangle }^{2}}/\langle R_{S}^{2}\left(t\right)\rangle$, has previously been used to define the critical shear rate indicated by dashed lines [14]. Subfigures (**a**,**b**) depict the grafted and the non-grafted scenario, respectively.

The motivation behind this definition is that the relative fluctuation of the squared extension in flow direction, defined as

(11) $$\sigma_{R_{S}^{2}}/R_{S}^{2}=\sqrt{\langle R_{S}^{4}\left(t\right)\rangle -{\langle R_{S}^{2}\left(t\right)\rangle }^{2}}/\langle R_{S}^{2}\left(t\right)\rangle ,$$

is maximal, when the probability of the bead-spring chain for changing from a collapsed to a non-collapsed state is the highest. For comparison with previous publications, we show $\sigma_{R_{S}^{2}}/R_{S}^{2}$ over $\dot{\gamma }$ in Figure 4.

**Figure 5:**
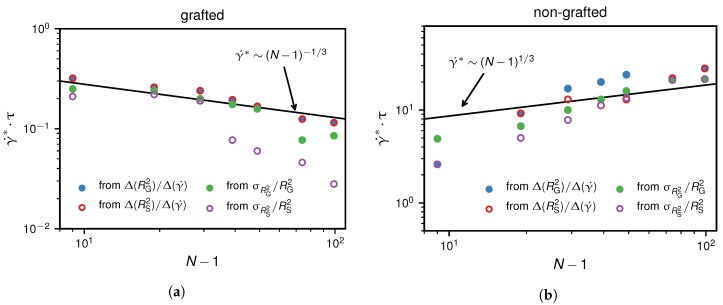
The critical shear rate, ${\dot{\gamma }}^{*}$, for the grafted and non-grafted scenarios as determined by four different criteria. We present the numerical derivatives and the normalized standard deviations of $R_{G}^{2}$ and $R_{S}^{2}$, indicated by dashed vertical lines in Figure 2 and Figure 4, as a function of N−1. The scaling predictions according to Equation (12) are shown as solid lines.

In addition to the relative fluctuations $\sigma_{R_{S}^{2}}/R_{S}^{2}$, we analysed several other quantities in terms of their dependence on the shear rate which we present in the supplementary material. In Figure 5, we compare the results for the critical shear rate as a function of *N* deduced from the maximum of the relative fluctuations and from the maximum of the numerical derivative of the two measures, $R_{S}^{2}$ and $R_{G}^{2}$, with respect to $\dot{\gamma }$. Note that the grafted and the non-grafted scenario show qualitatively different behaviours of the critical shear rate upon increasing the chain length. In the non-grafted case, an increase of the chain length leads to an increase in critical shear rate, whereas for the grafted scenario, an increase in chain length actually decreases the critical shear rate. Our results for the critical shear rate in the non-grafted case are in reasonable agreement with the previously derived scaling relation in the presence of hydrodynamic interactions, ${\dot{\gamma }}^{*}∼{\left(N-1\right)}^{1/3}$ [14]. From our simulation results, we find that the grafted scenario exhibits a different scaling according to ${\dot{\gamma }}^{*}∼{\left(N-1\right)}^{-1/3}$ which we show Figure 5a. This scaling can be explained by the fact that the anchor of the chain forms a protrusion, as soon as the cohesion force, $F_{C}∼\varepsilon /a∼k_{B}T/a$, can be overcome. Below the critical shear rate, the drag force acting on the globule is given by $F_{D}∼\dot{\gamma }{\left(N-1\right)}^{1/3}$ assuming that the drag force is proportional to the flow velocity at the position of the center of mass of the chain. Equating these two forces, we find ${\dot{\gamma }}^{*}∼{\left(N-1\right)}^{-1/3}$. Hence, we find the scaling laws

(12) $${\dot{\gamma }}^{*}∼\left\{\begin{array}{cc} {\left(N-1\right)}^{-1/3} & \left(\mathrm{HI},\mathrm{grafted}\right)\\ {\left(N-1\right)}^{1/3} & \left(\mathrm{HI},\mathrm{non-grafted}\right) \end{array}\right.,$$

which in Figure 5 are presented as solid lines and shown to describe the simulation data well.

**Figure 6:**
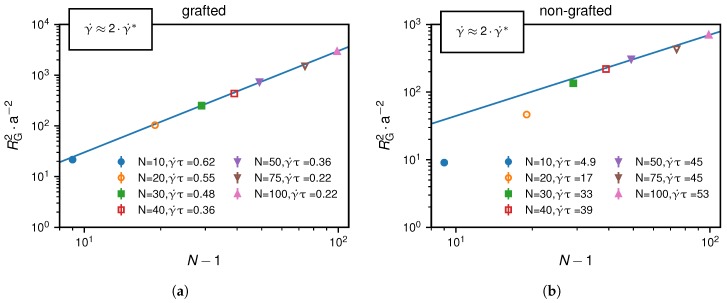
Squared radius of gyration slightly above the critical shear rate at $\dot{\gamma }\approx 2{\dot{\gamma }}^{*}$: The course of $R_{G}^{2}$ as a function of N−1 suggests for (**a**) the grafted scenario that the chain is fully stretched, $R_{G}^{2}∼N^{2}$, and (**b**) the non-grafted scenario that the chain is swollen, $R_{G}^{2}∼N^{6/5}$. The scaling relations shown as blue lines are given in Equation (13).

In Figure 6, we present $R_{G}^{2}$ at twice the critical shear rate in dependence of N−1. This characterizes the chain conformation slightly above the critical shear rate. We find a fully stretched conformation for the grafted scenario, while for the non-grafted case the conformation corresponds to a swollen polymer:

(13) $$R_{G}^{2}∼\left\{\begin{array}{cc} {\left(N-1\right)}^{2} & \left(\mathrm{at}\dot{\gamma }=2{\dot{\gamma }}^{*},\mathrm{grafted}\right)\\ {\left(N-1\right)}^{6/5} & \left(at\dot{\gamma }=2{\dot{\gamma }}^{*},\mathrm{non-grafted}\right) \end{array}\right..$$

We note that shear-induced transitions are usually referred to as globular-coil transitions [7,26,38]. However, in the grafted scenario the transition turns out to be rather a globular-stretch transition.

### 3.2. Tensile Force Profiles

The transition from a collapsed to a coiled or a stretched polymer upon increase of the shear rate is driven by a change in the tensile force profile inside the chain. The linear springs that connect the polymer beads act as force sensors for the tensile stress which counteracts the sum of shear stress and Lennard-Jones interactions. The absolute value of the distance between two beads along the chain contour determines the tensile force as

(14) $$f_{i}=\kappa \left(|r_{i+1}-r_{i}|-2a\right),\;i\in \left\{1,\ldots ,N-1\right\}.$$

Tensile forces shown in the following are always averaged over the course of a simulation by averaging the distance between consecutive beads.

**Figure 7:**
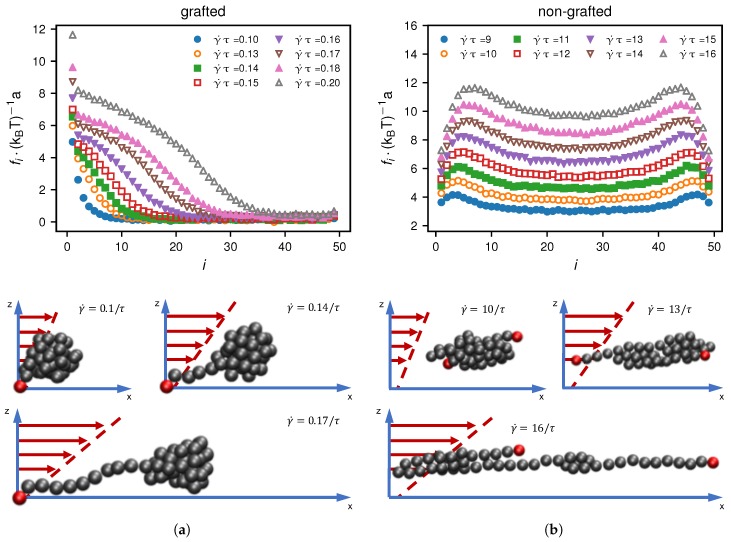
Comparison of tensile force profiles of a bead-spring chain with N=50 for different shear rates in the vicinity of the critical shear rate ${\dot{\gamma }}^{*}$ = 0.168 $\tau^{-1}$ for the (**a**) grafted and ${\dot{\gamma }}^{*}$ = 13 $\tau^{-1}$ for the (**b**) non-grafted case. These critical shear rates are based on the maximum of the numerical derivatives of $R_{S}^{2}$. We also present simulation snapshots that illustrate the different unfolding mechanisms in the grafted and non-grafted scenarios.

In Figure 7, we show the tensile force profiles in a small shear rate range around the critical shear rate for *N* = 50 of ${\dot{\gamma }}^{*}$ = 0.168 $\tau^{-1}$ in the grafted and ${\dot{\gamma }}^{*}$ = 13 $\tau^{-1}$ in the non-grafted scenarios. These tensile force profiles significantly differ between the grafted and non-grafted scenarios. The grafted case shows a maximum of the tensile force at the grafted monomer, followed by a monotonic decrease along the chain. Below the critical shear rate ${\dot{\gamma }}^{*}$ = 0.168 $\tau^{-1}$, most of the beads on average feel no tensile force. In the vicinity of the critical shear rate, the number of stretched bonds increases. What this shows is that depending on the shear rate, a subsection of the chain is elongated while the remaining part is still collapsed. In contrast, for the non-grafted case the maximum of the tensile force propagates towards the middle of the chain with increasing $\dot{\gamma }$, consistent with the protrusion mechanism for shear-induced unfolding introduced previously [7,14,26].

**Figure 8:**
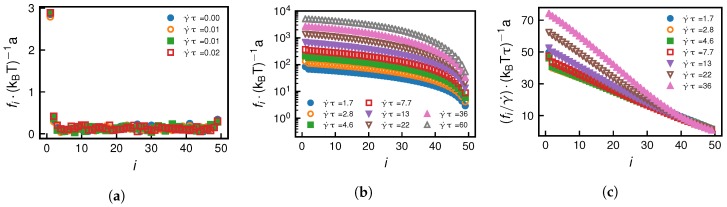
Grafted scenario: Tensile force profiles (N=50) for shear rates (**a**) far below and (**b**) far above the critical shear rate ${\dot{\gamma }}^{*}$ = 0.168 $\tau^{-1}$. In subfigure (**c**), we demonstrate the strong-stretching scaling collapse $f_{i}∼\dot{\gamma }$ predicted by Sing and Alexander-Katz [39]. Deviations from the scaling collapse coincide with a strong increase in mean radius of gyration shown in Figure 2a and are due to the extensible chain model.

In Figure 8a, we show the tensile force profiles for the grafted scenario far below the critical shear rate ${\dot{\gamma }}^{*}$ = 0.168 $\tau^{-1}$, which show no dependence on the shear rate, meaning that the shear stress is not sufficient to unfold parts of the chain. This observation is in line with $R_{G}^{2}$ not changing in that shear-rate regime (see Figure 2a). When $\dot{\gamma }$ becomes significantly larger than ${\dot{\gamma }}^{*}$, the tensile force profile takes a characteristic form, shown in Figure 8b, which has been studied in detail both theoretically and in simulations by Sing and Alexander-Katz [39]. Figure 8c demonstrates the predicted scaling relation, $f_{i}∼\dot{\gamma }$. Above a certain shear rate value, we see deviations from the strong stretching scaling $f_{i}∼\dot{\gamma }$, which is accompanied by a steep increase of $R_{G}^{2}$ with $\dot{\gamma }$ in Figure 2a and which is due to our usage of an extensible chain model.

### 3.3. Scaling of Lift and Drag for the Grafted Chain

**Figure 9:**
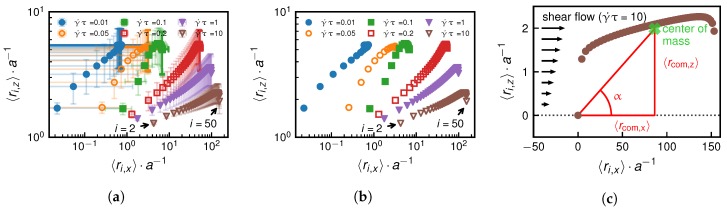
Grafted scenario: Subfigure (**a**) depicts the shear-rate-dependent mean position of individual beads of a chain with N=50 in the x−z plane together with the respective standard deviations. Subfigure (**b**) only shows the mean positions. The mean center of mass coordinates in *x* and *z* direction, $\langle r_{\mathrm{com},x}\rangle$ and $\langle r_{\mathrm{com},z}\rangle$, are exemplarily shown for the shear rate $\dot{\gamma }$ = 10 $\tau^{-1}$ in subfigure (**c**).

In Figure 9b, we show the mean positions of individual beads in the x−z plane for a chain with N=50 for different shear rates. In Figure 9a we also add the standard deviations, which demonstrates that the chain positions fluctuate significantly. To characterize the change in conformation of the bead-spring chain for the grafted case, we calculate the mean center of mass of the chain, which is defined as

(15) $$\langle r_{\mathrm{com}}\rangle =\frac{1}{N}\sum_{i=1}^{N}\langle r_{i}\rangle ,$$

and project it onto the plane of the shear flow.

**Figure 10:**
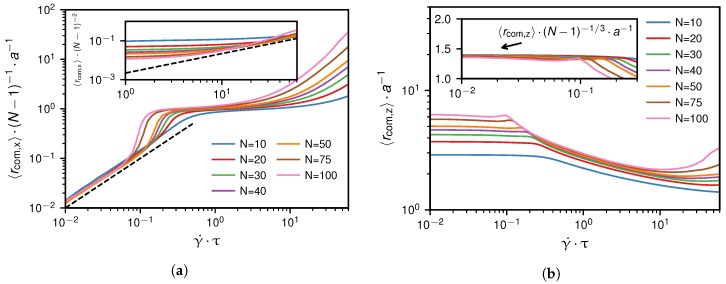
Grafted scenario: Subfigures (**a**,**b**) show the scaling collapse of $\langle r_{\mathrm{com},x}\rangle$ and $\langle r_{\mathrm{com},z}\rangle$ with respect to the monomer number as a function of the shear rate $\dot{\gamma }$ according to Equation (16) and (17). The dashed lines in subfigure (**a**) have slopes one and illustrate the proportionality to $\dot{\gamma }$.

We define the angle α between the wall and the center of mass as shown in Figure 9c. To determine the scaling of α with *N* in different shear rate regimes, we examine the scaling of the projected center of mass positions $\langle r_{\mathrm{com}},x\rangle$ and $\langle r_{\mathrm{com}},z\rangle$. Figure 10 shows simulation results from which we extract the following heuristic scaling relations,

(16) $$\langle r_{\mathrm{com}},x\rangle ∼\{\begin{array}{ll} \left(N-1\right)\;\dot{\gamma } & \left(\mathrm{below}\;{\dot{\gamma }}^{*}\right)\\ \left(N-1\right) & \left(\mathrm{above}\;{\dot{\gamma }}^{*}\right)\\ {\left(N-1\right)}^{2}\;\dot{\gamma } & \left(\mathrm{far}\;\mathrm{above}\;{\dot{\gamma }}^{*}\right) \end{array}$$

and

(17) $$\langle r_{\mathrm{com}},z\rangle ∼\{\begin{array}{ll} {\left(N-1\right)}^{1/3} & \left(\mathrm{below}\;{\dot{\gamma }}^{*}\right)\\ \mathrm{approximately}\;\mathrm{independent}\;\mathrm{of}\;\left(N-1\right)\;\mathrm{and}\;\dot{\gamma } & \left(\mathrm{above}\;{\dot{\gamma }}^{*}\right) \end{array}.$$

We discuss the scaling of the angle α by considering the geometric relation $\tan\left(\alpha \right)=\langle r_{\mathrm{com}},z/r_{\mathrm{com}},x\rangle$. Note that tan(α) directly relates to the force balance between the drag force, $F_{D}$, exerted on the chain by shear flow, and the hydrodynamic lift force, $F_{L}$.

**Figure 11:**
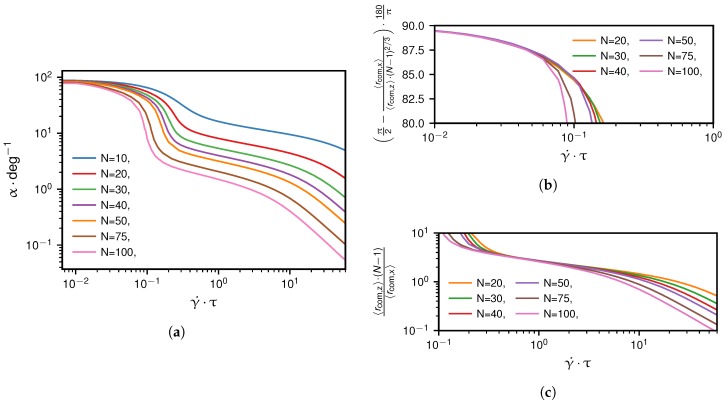
Grafted scenario: Subfigure (**a**) shows the dependence of the angle α, graphically defined in Figure 9c, on the chain length and on the shear rate. The scaling relations for the projections of the center of mass positions given by Equation (16) and (17) lead to the scaling of α below the critical shear rate, given by Equation (18) and shown in subfigure (**b**) and above the critical shear rate, given by Equation (19) and shown in subfigure (**c**).

Figure 11a shows the dependence of α on the shear rate. As long as that angle is much larger than $45^{\circ }$, which corresponds to the regime below the critical shear rate, we can use the relation arctan(x)≈π/2−1/x to deduce from Equation (16) and (17) the scaling of α as

(18) $$\alpha \approx \frac{\pi }{2}-\frac{\langle r_{\mathrm{com}},x\rangle }{\langle r_{\mathrm{com}},z\rangle }=\frac{\pi }{2}-\mathrm{const}\dot{\gamma }{\left(N-1\right)}^{2/3}\;\left(\mathrm{below}\;{\dot{\gamma }}^{*}\right),$$

which is confirmed in Figure 11b. This means that for low shear rates the change in angle α is governed by the drag force $F_{D}∼vR∼\dot{\gamma }{\left(N-1\right)}^{2/3}$.

When we consider shear rates slightly above the critical shear rate, where α becomes significantly smaller than $45^{\circ }$, we can use arctan(x)≈x, to derive the scaling given as

(19) $$\alpha \approx \frac{\langle r_{\mathrm{com}},z\rangle }{\langle r_{\mathrm{com}},x\rangle }∼{\left(N-1\right)}^{-1}\;\left(\mathrm{above}\;{\dot{\gamma }}^{*}\right),$$

which is confirmed in Figure 11c. For shear rates far above the critical shear rate, simulation results suggest the scaling $\alpha ∼{\left(N-1\right)}^{-2}{\dot{\gamma }}^{-1}$ which is deduced from Equation (16) and (17).

**Figure 12:**
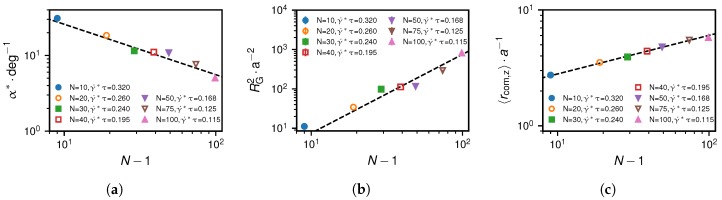
Grafted scenario at the critical shear rate ${\dot{\gamma }}^{*}$: Subfigure (**a**) depicts the simulation results for the angle α, subfigure (**b**) for the mean-square radius of gyration $R_{G}^{2}$ and subfigure (**c**) for the projection $\langle r_{\mathrm{com},z}\rangle$ depending on the monomer number, N−1. The deduced heuristic scaling relations which are indicated by dashed lines are $\alpha ∼{\left(N-1\right)}^{-2/3}$, $R_{G}^{2}∼{\left(N-1\right)}^{2}$ and $\langle r_{\mathrm{com},z}\rangle ∼{\left(N-1\right)}^{1/3}$.

In the following, we investigate the grafted chain system at the critical shear rate, which we indicate by an asterisk. In Figure 12a, we show the results for the dependence of the critical angle $\alpha^{*}$ on the monomer number, which is well described by the scaling relation $\alpha^{*}∼{\left(N-1\right)}^{-2/3}$. To derive this, we use that for the grafted scenario at the critical shear rate, the chain is already significantly stretched, i.e. we can use the same approximation for $\alpha^{*}$ as in Equation (19), $\alpha \approx \langle r_{\mathrm{com},z}/r_{\mathrm{com},x}\rangle$ which is equal to the ratio between lift and drag force magnitudes, $F_{L}/F_{D}$. Assuming Stokes’s law for the drag force, $F_{D}^{*}\approx 6\pi \eta R^{*}{\dot{\gamma }}^{*}{\langle r_{\mathrm{com},z}\rangle }^{*}$, where η is the viscosity, the scaling of ${\left(R_{G}^{2}\right)}^{*}$ and ${\langle r_{\mathrm{com},z}\rangle }^{*}$ in Figure 12b,c with the monomer number, given as ${\left(R_{G}^{2}\right)}^{*}∼{\left(N-1\right)}^{2}$ and ${\langle r_{\mathrm{com},z}\rangle }^{*}∼{\left(N-1\right)}^{1/3}$, and the dependence ${\dot{\gamma }}^{*}∼{\left(N-1\right)}^{-1/3}$ in Equation (12) allow us to deduce the dependence of the drag force at the critical shear rate on the chain length as

(20) $$F_{D}^{*}∼\left(N-1\right).$$

This relation arises due to the fact that the dependences of ${\dot{\gamma }}^{*}$ and ${\langle r_{\mathrm{com},z}\rangle }^{*}$ on N−1 cancel each other. When we combine the results for the scaling relations of $F_{D}^{*}$ and $\alpha^{*}$ in Equation (19) and (20), we find for the grafted scenario that the lift force at the critical shear rate is proportional to the mean *z*-position of the center of mass,

(21) $$F_{L}^{*}\approx \left(\alpha^{*}\;F_{D}^{*}\right)∼{\left(N-1\right)}^{1/3}.$$

A similar result has previously been derived by Sing and Alexander-Katz for the strong stretching limit [39] and apparently also is a good approximation at the critical shear rate. Note that the approximation becomes better for an increasing number of beads because the angle $\alpha^{*}$ decreases and we move closer to the strong stretching limit.

### 3.4. Comparison of Mean and Maximal Tensile Forces at Critical Shear Rate

Finally, we analyse the dependence of the tensile force profile in terms of its maximum and the mean value at the critical shear rate, defined as

(22) $$f_{\max}^{*}=\max_{i\in \{1,\ldots ,N-1\}}\left(f_{i}^{*}\right),$$

(23) $$f_{\mathrm{mean}}^{*}=\frac{1}{N}\sum_{i=1}^{N-1}f_{i}^{*}.$$

**Figure 13:**
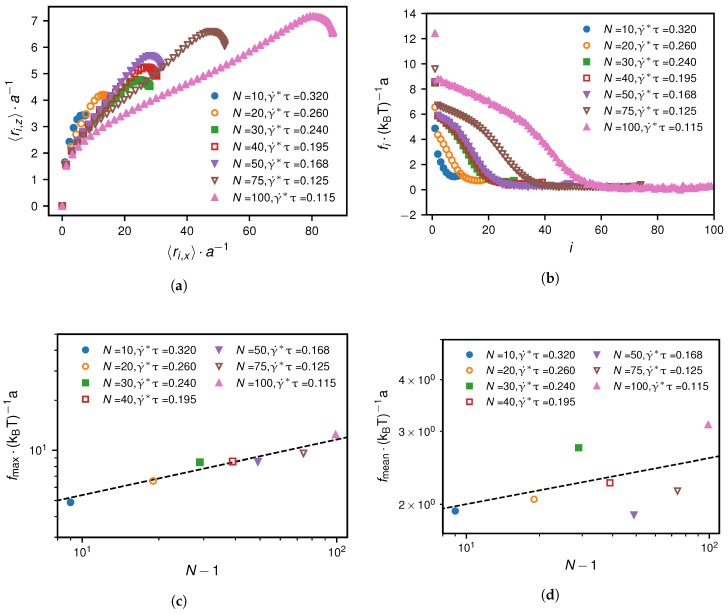
Grafted scenario at the critical shear rate ${\dot{\gamma }}^{*}$: Subfigure (**a**) shows the mean monomer positions 〈x〉(i) and 〈z〉(i). The resulting tensile force profiles are depicted in subfigure (**b**). Subfigures (**c**,**d**) show how the maximum and the mean of the tensile force profiles scale with the monomer number N−1. The simulation results suggest heuristic scaling laws, indicated as dashed lines, which are given in Equation(24) and (25).

Note that these quantities and their dependence on the number of monomers are relevant in relation to mechanosensitive folding and unfolding of VWF domains [19,27,28,29,30]. In Figure 13a, we show the mean positions of the beads for the grafted scenario at the critical shear rate. We analyse the corresponding tensile force profiles, shown in Figure 13b, in terms of their maximum values, shown in Figure 13c, and in terms of their mean values, shown in Figure 13d. The numerical results for the maximum and mean tensile forces for the grafted scenario at the critical shear rate suggest the following heuristic dependencies on the number of monomers:

(24) $$\begin{array}{cc} f_{\max}^{*} & ∼{\left({\dot{\gamma }}^{*}\right)}^{-1}∼{\left(N-1\right)}^{1/3}\;\left(\mathrm{grafted}\right), \end{array}$$

(25) $$\begin{array}{cc} f_{\mathrm{mean}}^{*} & ∼{\left({\dot{\gamma }}^{*}\right)}^{-1/3}∼{\left(N-1\right)}^{1/9}\;\left(\mathrm{grafted}\right). \end{array}$$

The small range of monomer numbers considered for the scaling law shown in Figure 13d limits the precision with which we are able to determine the scaling exponent. Hence, we would argue that the exponent we use in the heuristic law in Equation (25) is merely meant to be a satisfactory fit to the existing data. We find that for the grafted scenario at the critical shear rate, $f_{\max}^{*}$, which corresponds to the tensile force acting on the grafted monomer, is directly proportional to the radius of the collapsed chain while the mean tensile force becomes almost independent of the chain length, even though the drag force increases.

**Figure 14:**
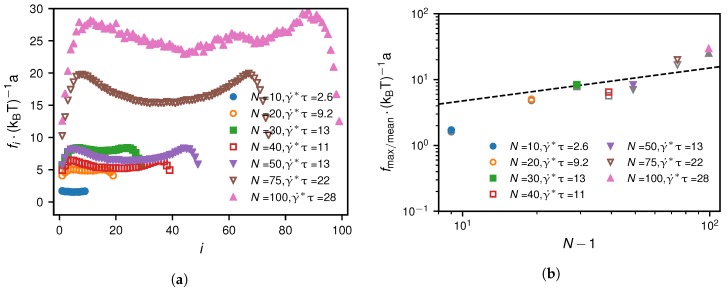
Non-grafted scenario at the critical shear rate ${\dot{\gamma }}^{*}$: Subfigure (**a**) shows the tensile force profiles at the critical shear rates for different monomer numbers. In subfigure (**b**) we show the maximum of the tensile force profiles in colour and the mean of the tensile force profiles in grey. For the non-grafted case, the maximum and mean tensile forces are very similar and are described by the same heuristic scaling given by Equation (26).

We also analyse the non-grafted scenario at the critical shear rate. The simulation results in Figure 14 for the tensile force profiles show that the maximum and the mean tensile forces are almost identical. Heuristically, we find for N≥20 the relation

(26) $$\begin{array}{cc} f_{\max/\mathrm{mean}}^{*} & ∼{\left({\dot{\gamma }}^{*}\right)}^{3/2}∼{\left(N-1\right)}^{1/2}\;\left(\mathrm{non-grafted}\right), \end{array}$$

which is shown to describe the simulation results well in Figure 14.

## 4. Conclusions

The grafted scenario for collapsed polymers under the influence of a constant linear shear flow exhibits very different unfolding behaviour compared to the scenario where the polymer is detached but in the vicinity of the vessel wall. Quantitatively, we find that the critical shear rate in the grafted case is inversely proportional to the size of the collapsed polymer. In comparison, we find the critical shear rate for the non-grafted scenario to increase proportionally with the size of the collapsed polymer which is in accordance to previous studies [6,7,14]. The general understanding is that shear activation of VWF is a key factor for the ability to bind blood platelets and thus to initiate the clot formation process and that the activation of VWF is accompanied by binding to exposed sub-endothelial collagen [3,7,19,20]. Hence, the two investigated scenarios are relevant cases to understand VWF’s involvement in coagulation especially when discussing possible cooperative effects. Recent studies have investigated the role of single domains of the VWF dimer in the activation process and their ability to bind to certain types of collagen [19,20,21]. Because there is only a preliminary understanding of the binding process and energies, cooperative effects are not at the focus of present VWF studies. However, when we put in physiological relevant parameters for the size of VWF (N=50 and a=73 nm [7,26]), we find that the critical shear changes from ${\dot{\gamma }}_{\mathrm{non-grafted}}^{*}=$ 11,000 Hz for the non-grafted scenario to ${\dot{\gamma }}_{\mathrm{grafted}}^{*}=140\;\mathrm{Hz}$ in the grafted scenario, where we use η=0.6913 mPa s for the dynamic viscosity of water at $37\;{}^{\circ }C$ [40]. By this consideration, we expect the attachment of VWF at the site of an injured vessel to considerably promote unfolding and to possibly lead to a cascade of activations of different sub-domains. While the shape of the tensile force profile significantly differs depending on the scenario, we show that the maximum of the tensile force profile at the critical shear rate is rather similar in both scenarios. By comparing the tensile force profiles for shear rates in the vicinity of the critical shear, we are able to illustrate the mechanisms responsible for unfolding in the two different scenarios. We reproduce the previously proposed protrusion mechanism [7,14,26] that governs the turn-over for the non-grafted scenario. We show that for the grafted scenario the mechanism is dominated by the tensile force acting on the grafted monomer and that unfolding of a subsection of the polymer, the size of which depends on the shear rate, characterizes the unfolding mechanism. Simulated tensile forces can be related to rupture forces of VWF domains from AFM measurements to estimate folding and unfolding times when we use transition rate theories [19,21,27,28,29,30]. Our analysis of the center of mass position dependence on the shear rate reveals the approximate angles at which the grafted polymer reaches into the solvent and allows for the determination of the relation between acting drag and lift forces. The reason for the opposite dependence of the critical shear rate on the size of the collapsed polymer originates in the distinctively different mechanisms that initiate unfolding. In the non-grafted scenario, protrusions have to build up and due to the rotational motion of the chain become wrapped around the chain [6,7,14]. Thus, the unfolding mechanism has been described as a nucleation process that depends on the sufficiently large size of the protrusions [6,14,26]. For the grafted scenario the chain cannot rotate since the first monomer is anchored. Hence, protrusions do not have to spontaneously occur in this scenario but are inherently present at all times for sufficiently large shear rates. Future work could investigate cooperative effects that might arise from a transition of the non-grafted scenario of VWF to the grafted scenario.

## Supplementary Materials

The following are available online at http://www.mdpi.com/2073-4360/10/8/926/s1, Figure S1: Grafted scenario: Comparison of different quantities which maximal value determines an estimate for the critical shear rate ${\dot{\gamma }}^{*}$, indicated by vertical dashed lines. Blue indicates that the quantity uses $R_{G}^{2}$, red uses $R_{S}^{2}$, Figure S2: Non-grafted scenario: Comparison of different quantities which maximal value determines an estimate for the critical shear rate ${\dot{\gamma }}^{*}$, indicated by vertical dashed lines. Blue indicates that the quantity uses $R_{G}^{2}$, red uses $R_{S}^{2}$, Table S1: Explicit simulation parameters for (a) the grafted and (b) the non-grafted scenario used in this study, Table S2: Grafted scenario: Comparison of the critical shear rate estimates in units of $\tau^{-1}$, determined as depicted in Figure 1 as the maximum value of the specific shear-rate dependent quantity, for different monomer numbers, Table S3: Non-grafted scenario: Comparison of the critical shear rate estimates in units of $\tau^{-1}$, determined as depicted in Figure S2 as the maximum value of the specific shear-rate dependent quantity, for different monomer numbers.

## Abbreviations

The following abbreviations are used in this manuscript:

VWF	von Willebrand factor
AFM	atomic force microscopy
HI	hydrodynamic interactions
com	center of mass
