# Supplementary material for: Hydrodynamic Shear Effects on Grafted and Non-Grafted Collapsed Polymers
Source: Polymers (Basel). 2018 Aug 18;10(8):926. doi: 10.3390/polym10080926 (PMC6403890; doi:10.3390/polym10080926)
Supplement: Supplementary file 1 [file polymers-10-00926-s001.pdf]

SUPPLEMENTARY MATERIAL
Hydrodynamic Shear Effects on Grafted and Non-Grafted Collapsed Polymers

Richard Schwarzl [1] and Roland R. Netz [1*]

[1] Department of Physics, Freie Universität Berlin, 14195 Berlin, Germany
[*] Correspondence: rnetz@physik.fu-berlin.de

1 Simulation Method

We motivate our over-damped Langevin equation by sketching how we arrive at the formula given in the main text,

$$\frac{\mathbf{r}_i(t+\Delta t)-\mathbf{r}_i(t)}{\Delta t} = \mu_0^{-1}\boldsymbol{\mu}_{\mathbf{ii}}\,\dot{\boldsymbol{\gamma}}\,\mathbf{r}_i(t) - \sum_{j=1}^{N}\boldsymbol{\mu}_{ij}\cdot\left[\nabla_{\mathbf{r}_j(t)}U(\mathbf{r}_1,...,\mathbf{r}_N)\right] + \mathbf{v}_i^{\text{correction}}(t) + \boldsymbol{\xi}_i(t). \tag{1}$$

The first term is the drag force introduced by the background flow field, $\mathbf{v}_\infty(z)$, without any disturbances. The velocity of the flow field is assumed to depend linearly on z as we want to describe a linear shear flow in x direction in vicinity of a no-slip boundary. The shear tensor is given as

$$\dot{\boldsymbol{\gamma}} = \begin{pmatrix} 0 & 0 & \dot{\gamma} \\ 0 & 0 & 0 \\ 0 & 0 & 0 \end{pmatrix} \tag{2}$$

The mobility tensor depending on the situation is either given by the Rotne-Prager-Yamakawa tensor (non-grafted)

$$\boldsymbol{\mu}_{ij} = \boldsymbol{\mu}^{\text{RPY}}\left(\boldsymbol{r}_{ij} = \boldsymbol{r}_i - \boldsymbol{r}_j\right) = \begin{cases} \frac{1}{8\pi\eta r_{ij}}\left[\left(1+\frac{2a^2}{3r_{ij}^2}\right)\mathbb{1} + \left(1-\frac{2a^2}{r_{ij}^2}\right)\hat{\mathbf{r}}_{ij}\otimes\hat{\mathbf{r}}_{ij}\right] & \text{if } r_{ij} > 2a \\ \frac{1}{6\pi\eta a}\left[\left(1-\frac{9r_{ij}}{32a}\right)\mathbb{1} + \frac{3r_{ij}}{32a}\hat{\mathbf{r}}_{ij}\otimes\hat{\mathbf{r}}_{ij}\right] & \text{if } r_{ij} \le 2a \end{cases}, \tag{3}$$

or by the Rotne-Prager-Blake tensor (grafted, no-slip at $z=0$)

$$\boldsymbol{\mu}_{ij} = \boldsymbol{\mu}^{\text{RPB}}\left(\boldsymbol{r}_i,\boldsymbol{r}_j\right) = \boldsymbol{\mu}^{\text{RP}}\left(\boldsymbol{r}_i - \boldsymbol{r}_j\right) - \boldsymbol{\mu}^{\text{RP}}\left(\boldsymbol{r}_i - \bar{\boldsymbol{r}}_j\right) + \boldsymbol{\Delta\mu}\left(\boldsymbol{r}_i,\boldsymbol{r}_j\right), \tag{4}$$

where $\bar{\boldsymbol{r}}_j = (x_j, y_j, -z_j)^{\text{T}}$ is the mirror image position and the Rotne-Prager tensor used in this definition is given as

$$\boldsymbol{\mu}^{\text{RP}}(\mathbf{r}) = \frac{1}{8\pi\eta r}\left(\mathbb{1} + \hat{\mathbf{r}}\otimes\hat{\mathbf{r}}\right) + \frac{a^2}{12\pi\eta r^3}\left(\mathbb{1} - 3\,\hat{\mathbf{r}}\otimes\hat{\mathbf{r}}\right). \tag{5}$$

The modification term arises from the Stokes and source doublets and has the following specific entries, as derived by von Hansen, et al. [1],

$$\boldsymbol{\Delta\mu}_{\alpha\alpha} = \frac{1}{4\pi\eta}\left[\frac{-z_i z_j}{R^3}\left(1-3\frac{R_\alpha^2}{R^2}\right) + \frac{a^2R_z^2}{R^5}\left(1-5\frac{R_\alpha^2}{R^2}\right)\right] \quad \text{, if } \alpha\in\{x,y\}, \tag{6}$$

$$\boldsymbol{\Delta\mu}_{zz} = \frac{1}{4\pi\eta}\left[\frac{z_i z_j}{R^3}\left(1-3\frac{R_z^2}{R^2}\right) - \frac{a^2R_z^2}{R^5}\left(3-5\frac{R_z^2}{R^2}\right)\right] \quad \text{, if } \alpha = z, \tag{7}$$

$$\boldsymbol{\Delta\mu}_{\alpha\beta} = \frac{1}{4\pi\eta}\left[\frac{3z_i z_j R_\alpha R_\beta}{R^5} - \frac{5a^2R_\alpha R_\beta R_z^2}{R^7}\right] \quad \text{, if } \alpha\in\{x,y\}, \alpha\neq\beta, \tag{8}$$

$$\boldsymbol{\Delta\mu}_{\alpha z} = \frac{1}{4\pi\eta}\left[\frac{z_j R_\alpha}{R^3}\left(1-3\frac{z_i R_z}{R^2}\right) - \frac{a^2R_\alpha R_z}{R^5}\left(2-5\frac{R_z^2}{R^2}\right)\right] \quad \text{, if } \alpha\in\{x,y\}, \tag{9}$$

$$\boldsymbol{\Delta\mu}_{z\alpha} = \frac{1}{4\pi\eta}\left[\frac{z_j R_\alpha}{R^3}\left(1+3\frac{z_i R_z}{R^2}\right) - 5\frac{a^2R_\alpha R_z^3}{R^7}\right] \quad \text{, if } \alpha\in\{x,y\}, \tag{10}$$

where $\mathbf{R} = \boldsymbol{r}_i - \bar{\boldsymbol{r}}_j$. For the limit of $\boldsymbol{r}_j$ going to $\boldsymbol{r}_i$, the Rotne-Prager-Blake tensor reduces to the self-mobility which is different for the directions parallel to the no-slip boundary and the perpendicular direction:

$$\boldsymbol{\mu}_{ii} = \boldsymbol{\mu}_{\text{self}}^{\text{RPB}}(z) = \lim_{r_{ij}\to 0}\boldsymbol{\mu}^{\text{RPB}}\left(\boldsymbol{r}_i,\boldsymbol{r}_j\right) = \begin{pmatrix} \mu_\parallel^{\text{RPB}}(z) & 0 & 0 \\ 0 & \mu_\parallel^{\text{RPB}}(z) & 0 \\ 0 & 0 & \mu_\perp^{\text{RPB}}(z) \end{pmatrix}, \tag{11}$$

where the parallel self-mobility is given by

$$\mu_{\parallel}^{\mathrm{RPB}}(z) = \frac{1}{6\pi\eta a}\left[1 - \frac{9a}{16z} + \frac{1}{8}\left(\frac{a}{z}\right)^3\right] + \mathcal{O}(a^4), \tag{12}$$

and the perpendicular self-mobility by

$$\mu_{\perp}^{\mathrm{RPB}}(z) = \frac{1}{6\pi\eta a}\left[1 - \frac{9a}{8z} + \frac{1}{2}\left(\frac{a}{z}\right)^3\right] + \mathcal{O}(a^4). \tag{13}$$

This symmetry-breaking leads to a correction term $\mathbf{v}_i^{\mathrm{correction}}$ that is given by [1]

$$\mathbf{v}_i^{\mathrm{correction}} = k_{\mathrm{B}}T \frac{\mathrm{d}\mu_{\perp}^{\mathrm{RPB}}(z)}{\mathrm{d}z}\bigg|_{z=z_i} \hat{\mathbf{z}}. \tag{14}$$

So consequently, the drag force defining the first term (drag term), depending on the situation, is given as

$$\mathbf{F}_{\mathrm{D},i} = \mu_0^{-1}\mathbf{v}_{\mathrm{i},\infty} = \begin{cases} \dot{\gamma}\ \mu_0\ z_i\ \hat{\mathbf{x}} & \text{(non-grafted)} \\ \dot{\gamma}\ \mu_{\parallel}^{\mathrm{RPB}}(z)\ z_i\ \hat{\mathbf{x}} & \text{(grafted)} \end{cases}. \tag{15}$$

The second term of Eq. (1) is the velocity of bead i due to the forces exerted by all other beads j. The last term of Eq. (1) follows from the fluctuation-dissipation theorem

$$\langle \boldsymbol{\xi}_i(t) \otimes \boldsymbol{\xi}_j(t') \rangle = 2k_{\mathrm{B}}T\boldsymbol{\mu}_{ij}\delta(t-t'). \tag{16}$$

To create random velocities that satisfy the correlation given by Eq. (16), we use the Cholesky decomposition of a matrix that is given as

$$\boldsymbol{\mu} = \begin{pmatrix} \boldsymbol{\mu}_{11} & \cdots & \boldsymbol{\mu}_{1N} \\ \vdots & \ddots & \vdots \\ \boldsymbol{\mu}_{N1} & \cdots & \boldsymbol{\mu}_{NN} \end{pmatrix} \tag{17}$$

which returns a lower triangular matrix, $\mathbf{L}$, that satisfies the relation $\mathbf{L}\,\mathbf{L}^{\mathrm{T}} = \boldsymbol{\mu}$. If we now define a normal random vector $\mathbf{x}$ that satisfies the conditions of gaussian white noise, meaning $\langle \mathbf{x}\,\mathbf{x}^{\mathrm{T}} \rangle = \mathbb{1}$, then one can write

$$\langle \boldsymbol{\mu} \rangle = \langle (\mathbf{L}\,\mathbf{x})\,(\mathbf{L}\,\mathbf{x})^{\mathrm{T}} \rangle = \langle \mathbf{L}\mathbf{x}\ \mathbf{x}^{\mathrm{T}}\mathbf{L}^{\mathrm{T}} \rangle = \mathbf{L}\langle \mathbf{x}\ \mathbf{x}^{\mathrm{T}} \rangle \mathbf{L}^{\mathrm{T}}. \tag{18}$$

So consequently, we used the following method to calculate the random velocities for each step:

$$\boldsymbol{\xi} = \begin{pmatrix} \boldsymbol{\xi}_1 \\ \vdots \\ \boldsymbol{\xi}_N \end{pmatrix} = \sqrt{\frac{2k_{\mathrm{B}}T}{\Delta t}}\,\mathbf{L}\,\mathbf{x}. \tag{19}$$

2 Simulation Parameters

In Tab. 1, we present explicit simulation parameters. For explanation, Δt is the chosen time step of a simulation, n_t is the total number of steps of a simulation and n_w is the number of steps that determines the period we use to write out positions of all beads.

N	$\Delta t/\tau$	n_t	n_w
10	0.0005	4000000000	100
20	0.0005	4000000000	1000
30	0.0005	1000000000	10000
75	0.0005	500000000	100
100	0.0005	500000000	10000

(a)

N	$\Delta t/\tau$	n_t	n_w
10	0.0005	4000000000	10000
30	0.0005	2000000000	10000
50	0.0005	1000000000	10000
75	0.0005	500000000	10000
100	0.0005	200000000	100000

(b)

Table 1: Explicit simulation parameters for (a) the grafted and (b) the non-grafted scenario used in this study.

3 Estimate Statistical Error of the Mean

Time dependent quantities such as the squared radius of gyration and the end to end distance in pull direction are analysed using the gromacs tool gmx analyze [2]. The error estimate of the time averaged quantity is calculated using the block average method according to the definition by Berk Hess [3],

$$\text{err.est.}(x(t)) = \sigma\sqrt{\frac{2(\alpha\tau_1 + (1-\alpha)\tau_2)}{T}}, \tag{20}$$

where σ is the standard deviation of the sample and $T = n_t\Delta t$ is the total time of the simulation run. The quantities τ_1, τ_2 and α are estimates for the short correlation time, the long correlation time and a weighting factor which are fitted using the auto correlation function of the observable in question. Note that we define the auto correlation function such that it decays to 0, meaning we actually look at the mean fluctuations of the observable minus its mean value over the sample,

$$C(\tau) = \frac{1}{(T-\tau)\sigma^2}\int_0^{T-\tau} [x(t) - \mu]\,[(x(t+\tau) - \mu]\,\mathrm{d}t. \tag{21}$$

A full derivation of the error estimate given in Eq. (20) can be found in the appendix of the 2002 article by Berk Hess [3].

4 Determining Critical Shear Rate

Determining the critical shear rate, $\dot{\gamma}^*$, is possible by evaluating any related statistical quantity that is affected by the change in shear rate and that becomes maximal or minimal at the point of transition between a collapsed to a non-collapsed state. Previous publications used the normalized fluctuations of the mean-square elongation R_S^2 of the chain in direction of the shear flow to pinpoint the transition [4, 5]. In the present study, we additionally investigate the mean-square radius of gyration R_G^2. We define the normalized standard deviation $\sigma_x = \sqrt{\langle x^2(t)\rangle - \langle x(t)\rangle^2 / \langle x(t)\rangle^2}$, where x is either R_G^2 or R_S^2. Fig. 1 and Fig. 2, we present results for the normalized standard deviation and three additional quantities of both R_G^2 and R_S^2 for the grafted and non-grafted scenario. The additional quantities are the error estimate of the mean determined as described in section 3, the numerical derivatives with respect to the shear rate which we refer to as $\Delta(R_\mathrm{G}^2)/\Delta(\dot{\gamma})$ and $\Delta(R_\mathrm{S}^2)/\Delta(\dot{\gamma})$ and the numerical derivative of the logarithmic values $\Delta\log(R_\mathrm{G}^2)/\Delta\log(\dot{\gamma})$ and $\Delta\log(R_\mathrm{S}^2)/\Delta\log(\dot{\gamma})$. The results for the critical shear rate for each quantity is presented in Tab. 2 for the grafted and in Tab. 3 for the non-grafted scenario. Judging from a comparison of individual plots in Fig. 1 and Fig. 2, we conclude that for our simulations the numerical derivatives $\Delta(R_\mathrm{G}^2)/\Delta(\dot{\gamma})$ and $\Delta(R_\mathrm{S}^2)/\Delta(\dot{\gamma})$ are the most consistent quantities to give the same critical shear rate value regardless whether we analyse the mean-squared radius of gyration or the mean-squared elongation in flow direction.

N	from $\sigma_{R_\mathrm{G}^2}/R_\mathrm{G}^2$	from err. est.$_{R_\mathrm{G}^2}/R_\mathrm{G}^2$	from $\Delta\log(R_\mathrm{G}^2)/\Delta\log(\dot{\gamma})$	from $\Delta(R_\mathrm{G}^2)/\Delta(\dot{\gamma})$	from $\sigma_{R_\mathrm{S}^2}/R_\mathrm{S}^2$	from err. est.$_{R_\mathrm{S}^2}/R_\mathrm{S}^2$	from $\Delta\log(R_\mathrm{S}^2)/\Delta\log(\dot{\gamma})$	from $\Delta(R_\mathrm{S}^2)/\Delta(\dot{\gamma})$
10	0.25	0.29	0.32	0.32	0.21	0.25	0.29	0.32
20	0.24	0.22	0.26	0.26	0.22	0.24	0.26	0.26
30	0.2	0.2	0.22	0.24	0.19	0.2	0.2	0.24
40	0.175	0.175	0.19	0.195	0.077	0.175	0.19	0.195
50	0.1575	0.06	0.1675	0.1675	0.06	0.1575	0.1675	0.1675
75	0.077	0.06	0.115	0.125	0.046	0.06	0.115	0.125
100	0.085	0.046	0.1	0.115	0.028	0.046	0.1	0.115

Table 2: Grafted scenario: Comparison of the critical shear rate estimates in units of τ^{-1}, determined as depicted in Fig. 1 as the maximum value of the specific shear-rate dependent quantity, for different monomer numbers.

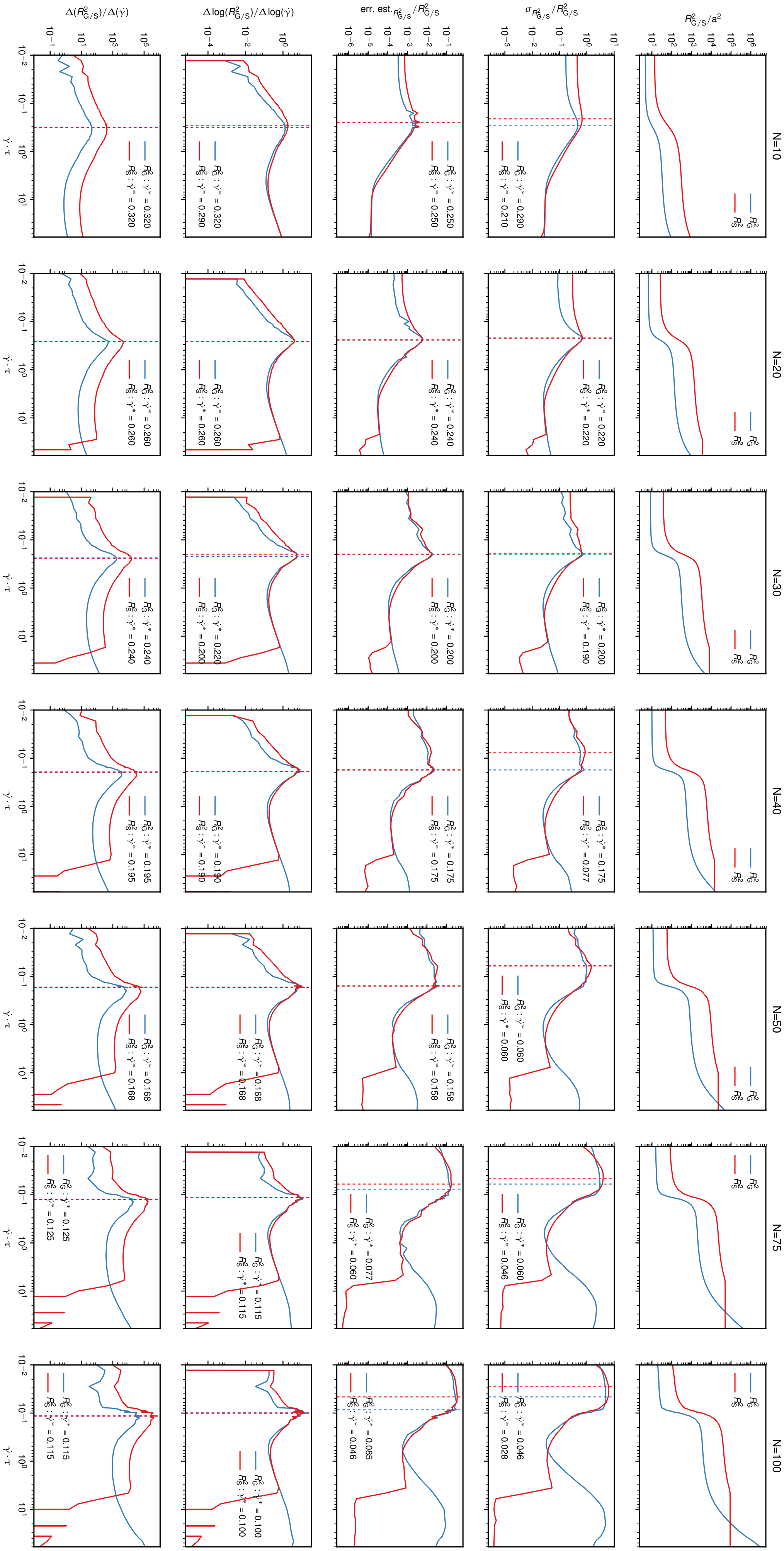

Figure 1: Grafted scenario: Comparison of different quantities which maximal value determines an estimate for the critical shear rate $\dot{\gamma}^*$, indicated by vertical dashed lines. Blue indicates that the quantity uses R^2_{G}, red uses R^2_{S}.

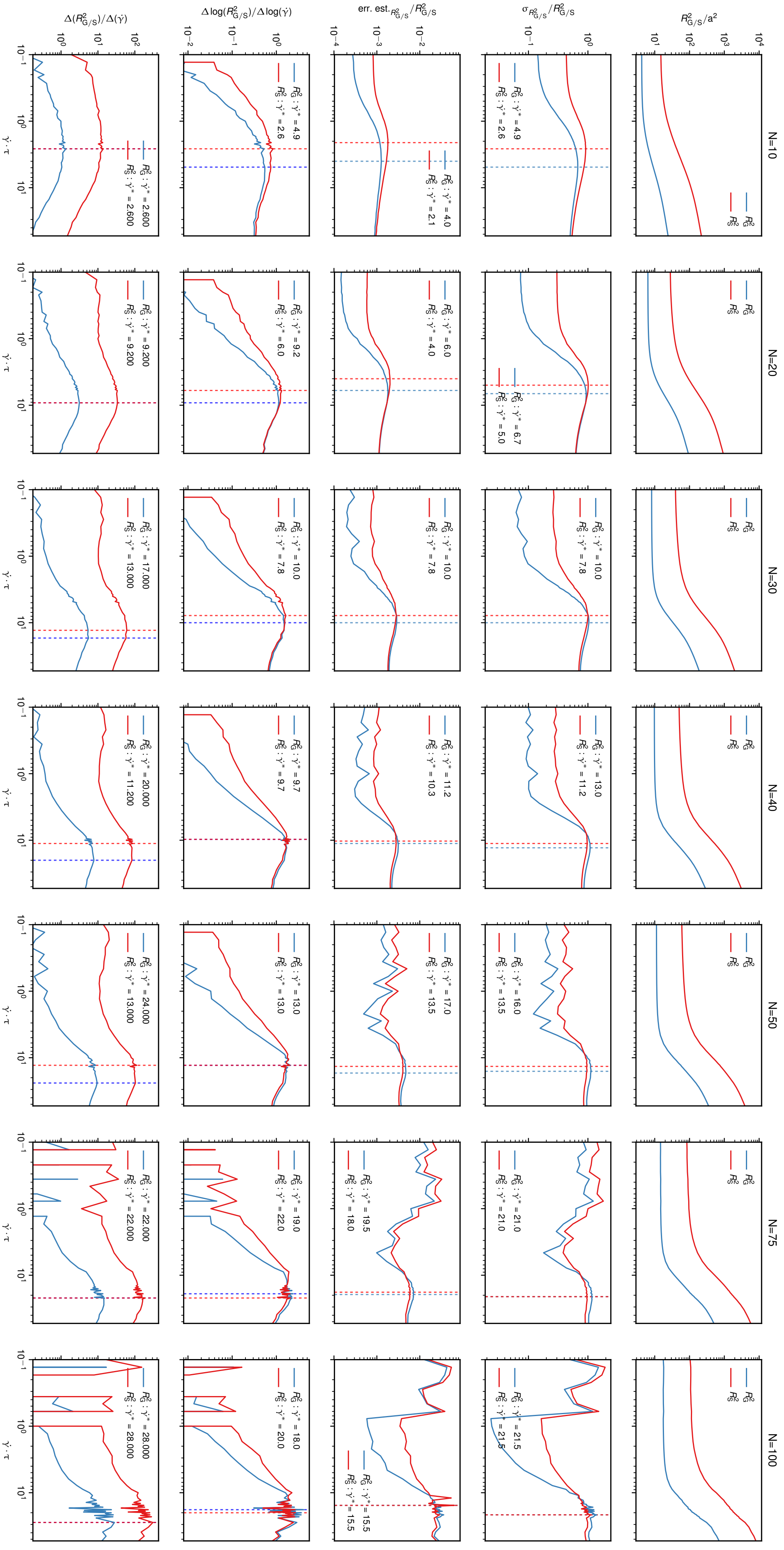

Figure 2: Non-grafted scenario: Comparison of different quantities which maximal value determines an estimate for the critical shear rate $\dot{\gamma}^*$, indicated by vertical dashed lines. Blue indicates that the quantity uses R_G^2, red uses R_S^2.

N	from $\sigma_{R_G^2}$/ R_G^2	from err. est.$_{R_G^2}$/ R_G^2	from $\Delta\log(R_G^2)$/ $\Delta\log(\dot{\gamma})$	from $\Delta(R_G^2)$/ $\Delta(\dot{\gamma})$	from $\sigma_{R_S^2}$/ R_S^2	from err. est.$_{R_S^2}$/ R_S^2	from $\Delta\log(R_S^2)$/ $\Delta\log(\dot{\gamma})$	from $\Delta(R_S^2)$/ $\Delta(\dot{\gamma})$
10	4.9	4	4.9	2.6	2.6	2.1	2.6	2.6
20	6.7	6	9.2	9.2	5	4	6	9.2
30	10	10	10	17	7.8	7.8	7.8	13
40	13	11.2	9.7	20	11.2	10.3	9.7	11.2
50	16	17	13	24	13.5	13.5	13	13
75	21	19.5	19	22	21	18	22	22
100	21.5	15.5	18	28	21.5	15.5	20	28

Table 3: Non-grafted scenario: Comparison of the critical shear rate estimates in units of τ^{-1}, determined as depicted in Fig. 2 as the maximum value of the specific shear-rate dependent quantity, for different monomer numbers.

References

[1] Y. von Hansen, M. Hinczewski, and R. R. Netz, *Hydrodynamic screening near planar boundaries: Effects on semiflexible polymer dynamics,* The Journal of Chemical Physics **134**, 235102 (2011).

[2] B. Hess, C. Kutzner, D. van der Spoel, and E. Lindahl, *GROMACS 4: Algorithms for Highly Efficient, Load-Balanced, and Scalable Molecular Simulation,* Journal of Chemical Theory and Computation **4**, 435 (2008).

[3] B. Hess, *Determining the shear viscosity of model liquids from molecular dynamics simulations,* The Journal of Chemical Physics **116**, 209 (2002).

[4] A. Alexander-Katz and R. R. Netz, *Dynamics and Instabilities of Collapsed Polymers in Shear Flow,* Macromolecules **41**, 3363 (2008).

[5] M. Radtke, S. Lippok, J. O. Rädler, and R. R. Netz, *Internal tension in a collapsed polymer under shear flow and the connection to enzymatic cleavage of von Willebrand factor,* The European Physical Journal E **39**, 32 (2016).
